# Social media interaction and built environment effects on urban walking experience: A machine learning analysis of Shanghai Citywalk

**DOI:** 10.1371/journal.pone.0320951

**Published:** 2025-04-29

**Authors:** Xingrui Chen, Yu Sun, Filzani Illia Binti Ibrahim, Myzatul Aishah Binti Kamarazaly, Siti Norzaini Binti Zainal Abidin, Suqiu Tang

**Affiliations:** 1 School of Architecture, Building and Design, Taylor’s University, Subang Jaya, Malaysia; 2 School of Art and Design, Jiangxi Institute of Fashion Technology, Xiangtang Development Zone, Nanchang City, Jiangxi Province, China; 3 School of Architecture Urban Planning Construction Engineering, Politecnico di Milano, Milano, Italy; Ain Shams University, EGYPT

## Abstract

In fast-paced urban environments, Citywalk has emerged as a key leisure activity for urban residents to alleviate stress and enhance emotional well-being. From the perspective of virtual-physical interaction, this study integrates social media data with geospatial information, utilizing machine learning methods and spatial statistical analysis to explore the multidimensional driving mechanisms and complex relationships affecting the emotional experiences of Citywalk participants. The findings indicate that the interaction index, as a core indicator of virtual social behavior, plays a key role in influencing emotional scores (SHAP value = 4.9104), exhibiting progressive effects without evident threshold characteristics. POI density demonstrates significant nonlinear threshold effects, with marginal benefits substantially increasing when density reaches 44.06. Additionally, spatial autocorrelation analysis of emotional scores reveals spatial clustering patterns, underscoring the critical role of interactions between virtual social behavior and physical spatial elements in emotional generation. In comparison, functional diversity and transit accessibility exhibit weaker but complementary effects on emotional scores. This research quantifies the roles of digital social behavior and the built environment in shaping emotional experiences from a virtual-physical interaction perspective, uncovering how virtual social behavior integrates into social space production through individual perception and social interaction. It extends theoretical frameworks in social space production and emotional geography. The findings provide data-driven insights for optimizing urban walking space design, proposing interaction index-oriented strategies to promote synergy between virtual and physical spaces, thus facilitating the creation of high-quality, emotionally friendly urban environments.

## Introduction

As urbanization continues to deepen, urban residents’ lifestyles and leisure preferences are undergoing rapid transformation [1]. People are gradually recognizing the key role of leisure activities in enhancing physical and mental well-being [2]. Against this backdrop, a non-commuting walking activity focused on experience and exploration—“Citywalk”—has quietly emerged in Chinese cities. Citywalk allows participants to engage in spontaneous and experiential walking behavior, enabling them to appreciate streetscapes and cultural atmospheres, thereby gaining pleasure and satisfaction [3,4]. This trend reflects urban residents’ desire for higher-quality walking environments and richer spatial experiences while presenting new challenges for urban planning and design.

Existing research predominantly focuses on traditional walkability analysis. In this field, numerous empirical studies have investigated how objective elements such as land-use mix, street connectivity, and destination accessibility influence daily commuting walking behavior [5–7]. However, these studies primarily address functional and utilitarian walking demands, such as commuting efficiency and convenience, with insufficient attention to perceptual experiences and emotional feedback during walking. The theoretical evolution of urban space understanding has progressed from an initial emphasis on physical morphology [8] to highlighting street life and social interaction [9], and further extending to place-making and emotional experience [10]. This theoretical trajectory demonstrates that high-quality walking environments must transcend basic functional requirements to address users’ subjective perceptions and social interaction needs. Within this context, the emerging phenomenon of Citywalk provides a unique analytical lens for understanding the multidimensional constitution of walking experiences.

Unlike commuting walks, Citywalk participants place greater emphasis on subjective feelings, aesthetic experiences, and emotional pleasure during their walks [11]. This emerging urban leisure activity demonstrates distinctive characteristics of virtual-physical interactions. At the physical level, participants acquire in-depth perception of street environments through slow-paced walking experiences. At the social level, the information sharing and interactive feedback mechanisms of social media platforms continuously reshape people’s emotional cognition of urban spaces. This highlights the necessity of incorporating subjective perception into walkability assessments, advocating for a more diversified and multidimensional understanding of walking activities. Traditional walking studies, largely based on objective indicators, often fail to fully capture walkers’ internal emotional responses and aesthetic preferences [12]. This highlights the necessity of incorporating subjective perceptions into walking environment assessments and calls for a more diverse and multifaceted understanding of walking activities.

From a theoretical perspective, research on Citywalk activities can be organically integrated into the analytical frameworks of social space production theory and sense of place theory. According to Lefebvre’s social space production theory [13], urban space is not a neutral physical background but rather a dynamic field shaped and reproduced by social practices, economic activities, cultural expressions, and individual experiences. With the proliferation of digital technologies, spatial production processes have become increasingly intertwined with virtual-physical interactions. Social media platforms serve as critical intermediaries that reshape individuals’ cognitive expectations of physical spaces, establishing distinctive information flow mechanisms [14]. These virtual social interactions intensify place-specific meanings and foster differentiated forms of place attachment [15]. This deep integration of virtual-physical interactions provides a new theoretical perspective for understanding the interaction between Citywalk participants and the environment.

Sense of place theory further emphasizes that urban spaces not only accommodate human behavior but also imbue spaces with unique social meaning through emotional connections, memories, and social interactions [16]. Erfani [17] indicates that place attachment forms through individuals’ subjective interpretation of environmental characteristics rather than objective attributes alone. On this basis, the continuous accumulation and dissemination of user-generated content (UGC) persistently reconstructs both the symbolic significance and usage patterns of physical spaces, generating dynamic behavioral feedback mechanisms [18]. This theoretical framework provides crucial support for analyzing how virtual social behavior influences the emotional experience of urban walking.

In recent years, the widespread application of digital technology has opened new dimensions for place-sense research. Cyberspace has become an important complement to physical places, with digital social behavior and virtual information flows profoundly influencing individual perceptions and emotional projections onto real spaces [19].

However, theoretical and methodological challenges remain. Traditional research methods (such as questionnaires and interviews) struggle to efficiently capture the dispersed and dynamic nature of Citywalk activities, while single regression models often fail to fully reveal nonlinear, complex spatial-emotional associations [20]. In contrast to traditional approaches, machine learning techniques, specifically neural networks and XGBoost algorithms, demonstrate remarkable advantages in identifying latent complex patterns within large-scale, multidimensional datasets and improving predictive accuracy [21–24]. Therefore, the introduction of big data and machine learning methods becomes necessary.

Addressing these issues and research gaps, this study proposes three core research questions: (a) How does virtual social behavior (quantified through interaction index) influence emotional experiences in Citywalk activities? (b) How do built environment elements (such as POI density) interact with virtual social behavior to shape emotional experiences? (c) What are the spatial differentiation characteristics of emotional experiences and their underlying social space production mechanisms? Through systematically addressing these questions, this research aims to reveal the formation mechanisms of urban walking experiences in the digital era and deepen our understanding of virtual-physical interaction pathways.

The innovations of this study are manifested in three aspects: First, from a theoretical perspective, it constructs a virtual-physical interaction assessment framework, extending social space production theory into the digital era, thus providing new insights into understanding the influence mechanisms of virtual social behavior. Second, from a methodological perspective, it develops machine learning approaches for multi-source data analysis, revealing the interactive characteristics between built environment elements and virtual social behavior. Third, from a practical perspective, it proposes interaction index-oriented spatial optimization strategies based on spatial differentiation characteristics, supporting the creation of high-quality urban walking spaces. These innovations not only deepen our understanding of Citywalk activities but also provide operational references and guidance for future urban space design and policy-making.

## Methodology

**Fig 1:**
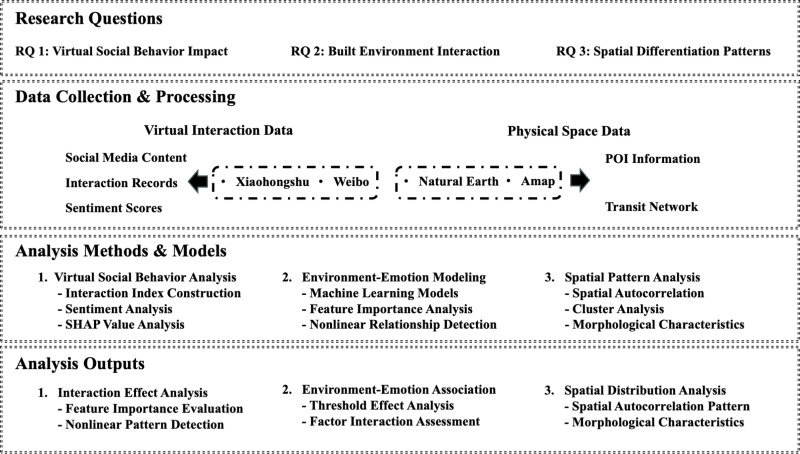
Research framework for analyzing virtual-physical interaction in Citywalk activities.

To systematically reveal the impact mechanism of virtual-physical interaction on the emotional experience of urban walking, this study establishes a multi-level analytical framework (Fig 1). This framework is based on three core research questions and integrates virtual interaction data (social media content, interaction records, emotional scores) with physical spatial data (POI information, transportation networks). By applying multidimensional analytical methods such as machine learning modeling and spatial statistics, the framework ultimately achieves a comprehensive evaluation of the impact of virtual social behavior, environment-emotion associations, and spatial differentiation characteristics.

### Social media and geographic data collection

This study focuses on citywalk activities in Shanghai, utilizing an integrated framework of social media and geographic information data. Shanghai’s complex urban spatial structure and diverse leisure activities make it an ideal region for studying citywalk behavior, with its high-density public space layout, diversified urban functional zones, and advanced public transit system providing strong representativeness and research value [25]. Additionally, as a leading Asian metropolis, Shanghai’s Citywalk popularity and participation patterns demonstrate universality, supporting the generalization of research findings.

The social media data used in this study were sourced from two platforms: Xiaohongshu and Weibo. Xiaohongshu is known for its experience-based sharing with rich text and images, while Weibo is characterized by its immediacy and extensive user base [26]. Additionally, Citywalk-related content on these platforms demonstrates high user engagement and geographic information density. Given the specificity of the research topic, the primary search term “Shanghai Citywalk” was used, and to account for the diversity of user expressions, variant forms such as “Shanghai city walk” and “Modu Citywalk” were also included to ensure comprehensive data collection.

The data collection period spanned from August 2023 to August 2024. To ensure data quality and representativeness, a multi-level filtering strategy was adopted. After data cleaning, advertisements, machine-generated content, and duplicate posts were removed, resulting in 23,436 valid samples out of 31,582 original entries. Furthermore, based on engagement indicators—including the number of likes, shares, and comments—posts with zero engagement were excluded to ensure that the selected samples reflect substantive social influence. This filtering strategy not only guarantees data quality but also accurately represents real social participation.

Geographic information extraction employed a combination of manual annotation and named entity recognition techniques. From the cleaned samples, 5,000 posts were randomly selected for annotating geography-related entities such as shops, attractions, streets, and squares. These annotated data were used to fine-tune the RoBERTa-wwm-ext model for automatic geographic entity extraction from remaining posts [27], ultimately yielding 4,010 POIs. These POIs cover diverse spatial entities, providing critical data for subsequent analysis.

Each POI’s functional attributes (e.g., dining, shopping, culture) and WGS84 geographic coordinates were obtained through the Amap API. This process revealed spatial distribution characteristics of citywalk activities while establishing foundations for indicator construction and model analysis. Additionally, administrative divisions, road networks, and public transit facility (bus stops, metro stations) spatial distribution data were obtained from OpenStreetMap. By combining these data sources with Amap POI data and land use data, this study comprehensively and systematically characterized urban functional layout and diversity.

### Data integration and grid construction

A grid-based data aggregation strategy was adopted to systematically analyze citywalk activity spatial distribution and its relationship with built environment factors. Shanghai’s entire territory was divided into 7,470 grid cells, each measuring 1 km × 1 km. This scale selection referenced satellite data resolution and common urban research applications, ensuring broad regional coverage and analytical convenience [28–31]. Based on citywalk-related POI spatial distribution, only 880 grid cells contained valid POI information. Grids without any relevant POIs were excluded from further statistical analysis to ensure data accuracy and relevance.

Within valid grid cells, two key indicators were calculated: POI density and functional diversity. POI density measures spatial concentration of citywalk-related POIs, while functional diversity is quantified using the Shannon entropy index:


$$H=-\sum_{i=1}^{n}p_{i}\ln p_{i}$$

where *p*_*i*_ represents the proportion of POI type *i* within the grid, and n is the total number of POI types. Higher Shannon entropy values indicate more diverse functional structures.

To evaluate public transit accessibility’s impact on citywalk activities, this study combined spatial data of bus and metro stations to calculate transit convenience for each grid. Referencing fuzzy Delphi and fuzzy analytic hierarchy process results, original weights for metro and bus were 0.254 and 0.134 respectively [32]. After standardization considering only these two transit modes, metro weight became 0.655 and bus weight 0.345. Based on this, the transit convenience indicator is defined as:


$$T=0.655\cdot {\bar{d}}_{metro}+0.345\cdot {\bar{d}}_{bus}$$

where $\overset{―}{d}$_*metro*_ and $\overset{―}{d}$_*bus*_ represent average distances from grid POIs to nearest metro and bus stations respectively. Lower *T* values indicate higher grid transit accessibility.

### Sentiment Analysis

Text content from social media posts underwent sentiment analysis, aimed at evaluating Citywalk participants’ emotional experiences. The HanLP model was employed [33], with domain-specific adaptation for citywalk-related vocabulary.

After completing sentiment scoring for all posts, scores were aggregated to POI level. Considering multiple mentions of the same POI, each POI’s sentiment score was calculated as a weighted average:


$$Sen_{i}=\frac{\sum_{j=1}^{n_{i}}Sen_{j}\cdot w_{ij}}{\sum_{j=1}^{n_{i}}w_{ij}}$$

where *Sen*_*i*_ represents POI *i*’s comprehensive sentiment score, *Sen*_*j*_ is the sentiment score of post *j* mentioning POI *i*, *w*_*ij*_ is the mention frequency of POI *i* in post *j*, and *n*_*i*_ is the total number of posts mentioning POI *i*.

POI sentiment scores were further aggregated to grid level, calculating grid sentiment scores as arithmetic means of contained POIs:


$$SEN_{k}=\frac{1}{m_{k}}\sum_{i=1}^{m_{k}}Sen_{i}$$

where *SEN*_*k*_ represents grid *k*’s comprehensive sentiment score, and *m*_*k*_ is the total number of POIs in grid *k*. Grid sentiment scores served as the core dependent variable for subsequent environment-emotion association analysis.

### Interaction Analysis

Social media interaction intensity is a key dimension for understanding citywalk activity popularity and social influence. Interaction behaviors such as likes, shares, and comments directly reflect participant preferences and interests [34], serving as important indicators for evaluating citywalk experiences. This study constructed POI interaction intensity indicators based on social media interaction data, aggregating to grid level to characterize socialized participation patterns in citywalk activities.

First, three types of interaction data (likes, shares, comments) for each citywalk-related post underwent standardization. Referencing literature on interaction behavior influence [35], weights of 0.2, 0.3, and 0.5 were assigned to likes, shares, and comments, respectively, based on their relative importance in reflecting engagement. Post comprehensive interaction scores were calculated as:


$$Int_{j}=0.2\cdot \frac{L_{j}}{L_{\max}}+0.3\cdot \frac{S_{j}}{S_{\max}}+0.5\cdot \frac{C_{j}}{C_{\max}}$$

where *Int*_*j*_ represents post *j*’s comprehensive interaction score, *L*_*j*_, *S*_*j*_, and *C*_*j*_ represent likes, shares, and comments quantities respectively, and *L*_*max*_, *S*_*max*_, *C*_*max*_ represent sample maximum values.

Then, POI comprehensive interaction index Inti was calculated based on multiple mention scenarios. Weighted averaging was adopted to balance different posts’ contributions:


$$Int_{i}=\frac{\sum_{j=1}^{n_{i}}Int_{j}\cdot w_{ij}}{\sum_{j=1}^{n_{i}}w_{ij}}$$

where Inti represents POI *i*’s interaction index, *w*_*ij*_ is POI *i*’s mention frequency in post *j*, and *n*_*i*_ is the total number of posts mentioning POI *i*.

Finally, POI interaction indices were aggregated to the grid level by calculating the grid interaction index as the arithmetic mean of all POI interaction indices within the grid:


$$INT_{k}=\frac{1}{m_{k}}\sum_{i=1}^{m_{k}}Int_{i}$$

where *INT*_*k*_ represents grid *k*’s comprehensive interaction index, and *m*_*k*_ is the total number of POIs in grid *k*. This indicator reflects socialized participation level of citywalk activities within grid scope, forming the basis for analyzing relationships between activity popularity and built environment.

### Model development and evaluation

This study extracted multidimensional features including POI density, Shannon entropy index, transit accessibility, and social media interaction intensity for each 1-square-kilometer grid cell, with grid-level aggregated sentiment scores as dependent variables. To balance model fitting and generalization capabilities, the dataset was divided into 704 grids (80%) for training and 176 grids (20%) for testing. This ratio balances model fitting and generalization ability while ensuring result robustness and reproducibility [36].

Three representative machine learning algorithms were selected for comparative evaluation: multilayer perceptron (MLP) neural network, random forest (RF), and gradient boosting decision tree (XGBoost). These models were chosen based on their excellence in handling complex nonlinear relationships (MLP), robustness to high-dimensional data (RF), and gradient boosting algorithm optimization (XGBoost) [37,38]. Model performance was evaluated using root mean square error (RMSE), coefficient of determination (R²), and mean R² with standard deviation under 5-fold cross-validation [39]. For hyperparameter tuning, optimization strategies like GridSearchCV were employed to obtain optimal parameter combinations for each model.

To deeply understand influence mechanisms behind model predictions, this study further introduced SHAP method to quantitatively analyze feature importance for sentiment scores [40]. By comparing SHAP values across different features, contribution differences in model predictions were revealed, thereby inferring inherent action logic between built environment elements and citywalk emotional experiences. This game theory-based feature importance analysis approach compensates for traditional regression coefficient limitations, providing new perspectives for black-box model interpretability [41].

## Result

### Descriptive statistics and spatial distribution characteristics

This study revealed spatial distribution characteristics of citywalk activities through grid-scale statistical analysis.

**Fig 2:**
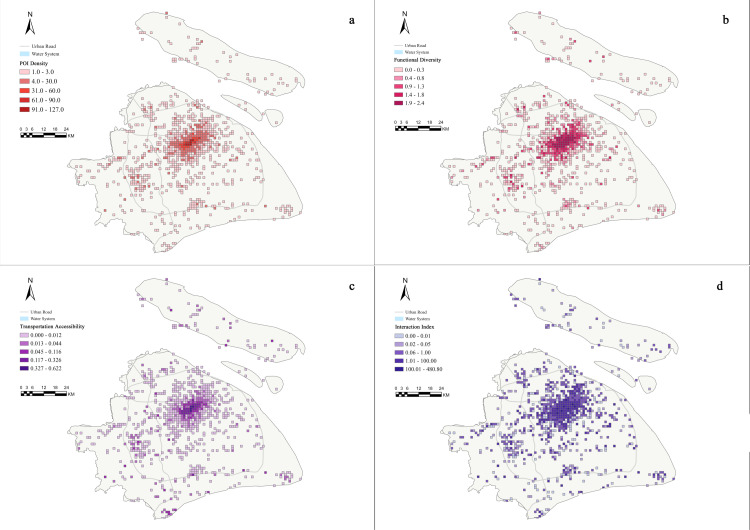
Spatial distribution of key indicators for Citywalk activity in Shanghai (a) POI density (points per grid); (b) functional diversity (Shannon index); (c) transportation accessibility; (d) interaction index. The base map data were obtained from Natural Earth (https://www.naturalearthdata.com/).

Regarding POI density, distribution across 880 valid grid cells showed significant imbalance. The median POI count was 1, with an average of 4.56 POIs per grid (SD = 12.77). Maximum density reached 127 POIs, while 50% of grids contained no more than 1 POI. High-density areas (91–127 POIs) were primarily concentrated at the intersection of Xuhui, Jing’an, Changning, and Huangpu districts, showing a significant reduction in POI density toward peripheral areas (31–90 POIs, Fig 2a).

In terms of functional composition, the 4,010 POIs encompassed 14 major functional types, with shopping and market POIs accounting for the highest proportion (16.96%), followed by comprehensive dining (11.77%) and natural/outdoor facilities (11.37%). Cultural facilities represented a considerable share (13.34%), while transportation facilities constituted 9.30% (Fig 2b). High functional diversity areas (Shannon entropy 1.9–2.4) were predominantly located in regions with high POI density, emphasizing their spatial correlation (Fig 2b).

Transit convenience indicators showed grid-scale distribution ranging from 0 to 0.622, with a mean of 0.018 (SD = 0.053). Seventy-five percent of grids exhibited transit convenience values below 0.012, while high-value areas (above 0.012) were predominantly concentrated in core districts, including the intersections of central urban zones (Fig 2c).

Interaction indices showed broad distribution (0–480.805), averaging 4.447 (SD = 22.342). Seventy-five percent of grids had interaction indices below 1.043, while only a few grids reached values above 100.01 (Fig 2d).

**Fig 3:**
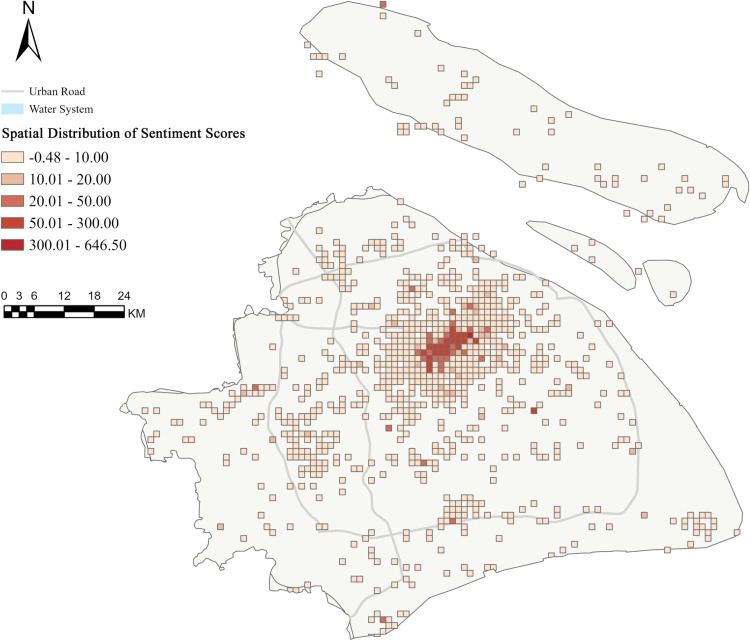
Spatial distribution of Citywalk sentiment scores across Shanghai’s urban districts. Base map source: Natural Earth.

Sentiment scores similarly exhibited spatial heterogeneity. Grid sentiment scores averaged 6.686 (SD = 30.664), ranging from −0.487 to 646.501. The median sentiment score was 0.620, while 75% of grids scored below 2.696. High sentiment score areas (300.01–646.50) were concentrated in high-density regions, with peripheral areas predominantly showing low scores (−0.48–20.00) (Fig 3).

### Model performance evaluation and feature importance analysis

Machine learning methods were employed to explore key factors influencing citywalk emotional experiences. Ensuring result robustness, 880 samples were divided into training (704) and test (176) sets, with 5-fold cross-validation assessing generalization capability. Table 1 reports performance comparisons among neural network, random forest, and XGBoost models. Neural networks demonstrated optimal test set performance (R²=0.8470, RMSE=5.0970) and stable generalization in cross-validation (CV Mean R²=0.8527, Std=0.0665). Comparatively, random forest and XGBoost showed lower test set R² by 7.3% and 6.8% respectively, with greater cross-validation fluctuations (Std=0.1374 and 0.1009 respectively).

**Table 1:** Model comparison results.

Model	Test RMSE	Test R²	CV Mean R²	CV Std R²
Neural Network	5.0970	.8470	.8527	.0665
Random Forest	6.0419	.7851	.7801	.1374
XGBoost	5.9741	.7899	.7803	.1009

**Fig 4:**
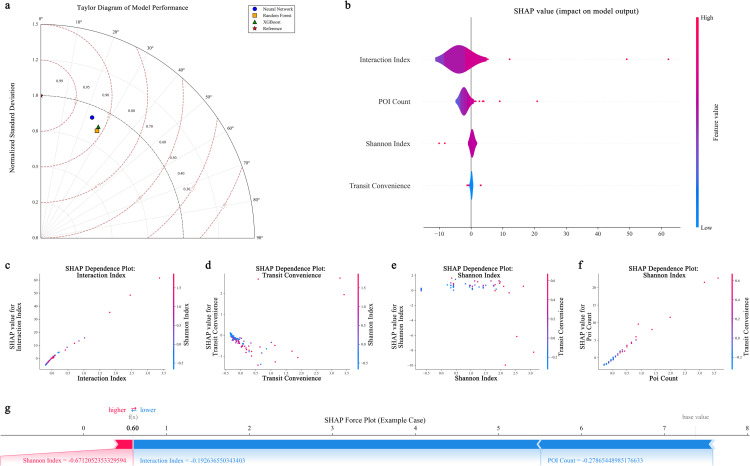
Model performance evaluation and feature importance analysis: (a) Taylor diagram comparing predictive performance of neural network (●), random forest (□), and XGBoost (△) models; (b) SHAP summary plot showing magnitude and distribution of feature impacts on sentiment score predictions; (c-f) Dependence relationships; (g) Force plot analysis.

To comprehensively evaluate and interpret the model, a series of analyses were conducted (Fig 4). The Taylor diagram (Fig 4a) demonstrated neural networks’ distinct advantages in both standard deviation and correlation coefficient dimensions. The SHAP summary plot (Fig 4b) revealed the magnitude of feature impacts, with interaction index and POI count showing mean SHAP values of 4.9104 and 2.4119 respectively, substantially higher than Shannon index (0.5550) and transit convenience (0.2575). The SHAP dependence plots (Fig 4c–4f) further illustrated the nonlinear impact patterns of each feature, while the SHAP force plot (Fig 4g) provided feature contribution analysis for a specific case.

### Feature importance and nonlinear relationships

Piecewise regression revealed nonlinear relationships between built environment characteristics and citywalk sentiment scores (Table 2). Interaction index showed highest goodness-of-fit (R²=0.9422) without obvious threshold points, suggesting progressive influence. Conversely, POI quantity (R²=0.5500), transit convenience (R²=0.5151), and Shannon index (R²=0.3545) exhibited significant threshold characteristics. For POI quantity, impact on sentiment scores significantly strengthened at density of 44.0553.

**Table 2:** Piecewise regression results.

Feature	R²	Threshold Point
POI Quantity	.5500	44.0553
Shannon Index	.3545	.6799
Transit Convenience	.5151	.2095
Interaction Index	.9422	**–**

Correlation analysis revealed significant interaction effects among built environment characteristics (Table 3). Interaction index showed extremely strong positive correlation with Shannon index (r=0.9594, p<0.001), suggesting functional diversity might indirectly influence sentiment scores through promoting social interaction. Correlation between interaction index and POI quantity (r=0.9224, p<0.001) further supported observations of high-density places fostering positive social atmospheres.

**Table 3:** Feature interaction analysis results.

Feature Combination	Correlation Coefficient	P-value	Significance
POI Quantity × Shannon Index	.609	.000	***
POI Quantity × Transit Convenience	.934	.000	***
POI Quantity × Interaction Index	.922	.000	***
Shannon Index × Transit Convenience	.660	.000	***
Shannon Index × Interaction Index	.959	.000	***
Transit Convenience × Interaction Index	.867	.000	***

**Fig 5:**
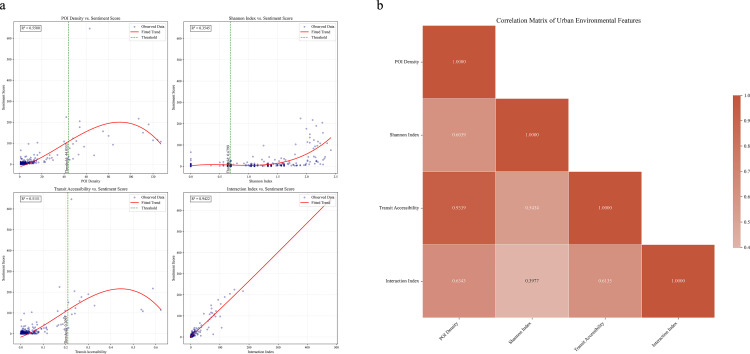
Nonlinear relationships between environmental features and sentiment scores: (a) Piecewise regression scatter plots showing threshold effects; (b) Correlation heatmap of environmental features.

Fig 5 illustrates the nonlinear relationships between environmental features and sentiment scores, as well as inter-feature correlations. The piecewise regression scatter plots (Fig 5a) reveal that both POI density and transit convenience exhibit distinct trend changes around their threshold points, demonstrating inverse U-shaped nonlinear characteristics. The relationship between Shannon index and sentiment scores shows an upward trend after the threshold point, while the interaction index displays a more pronounced linear positive correlation pattern. The fitted curves clearly demonstrate these nonlinear characteristics. The correlation heatmap (Fig 5b) quantifies the strength of associations between features, with POI density and transit convenience showing the strongest positive correlation (r=0.9339), while the Shannon index demonstrates relatively weaker correlations with other features.

### Spatial patterns and autocorrelation analysis

**Fig 6:**
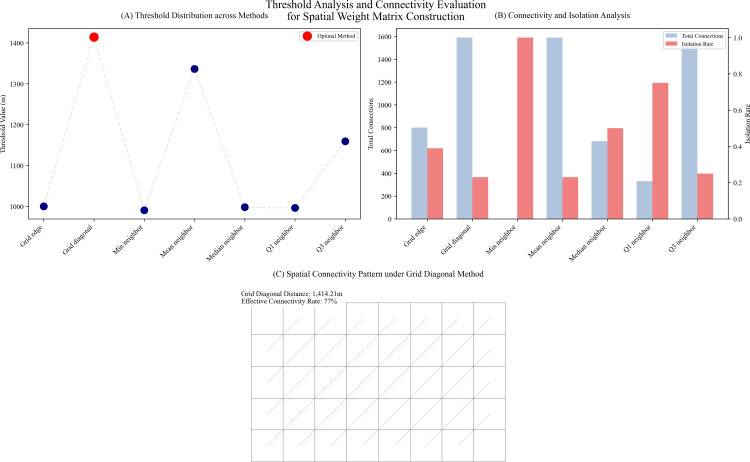
Threshold analysis of the spatial weight matrix. (A) Comparison of threshold distributions; (B) Connectivity analysis; (C) Spatial connectivity pattern of the grid diagonal method.

This study employs global and local spatial autocorrelation metrics to systematically analyze the geographic distribution patterns of Citywalk sentiment scores. First, by comparing various distance threshold calculation methods (Fig 6, Table 4), this study identified a spatial weight matrix construction scheme based on the grid diagonal distance (1,414.21 meters). As shown in Fig 6A, different threshold methods produce varying distance values, among which the grid diagonal method demonstrates the best performance. Connectivity analysis (Fig 6B) indicates that this method effectively balances grid unit spatial proximity while ensuring connectivity, with an isolation rate of 23%, exhibiting greater robustness and analytical applicability compared to other threshold methods. The spatial connectivity pattern under the grid diagonal method (Fig 6C) presents an effective network structure with a connectivity rate of 77%.

**Table 4:** Threshold analysis and connectivity evaluation results.

Method	Threshold	Total Connections	Average Connections	Isolated Grids	Isolation Rate
Grid edge	1,000.00	800.00	.91	344.00	.39
Grid diagonal	1,414.21	1590.00	1.81	204.00	.23
Min neighbor	990.32	2.00	.00	878.00	1.00
Mean neighbor	1,336.38	1588.00	1.80	205.00	.23
Median neighbor	997.73	680.00	.77	440.00	.50
Q1 neighbor	996.30	332.00	.38	660.00	.75
Q3 neighbor	1,159.41	1548.00	1.76	220.00	.25

**Note:** The isolation rate represents the proportion of grid units with no spatial connections under the respective threshold. The grid diagonal method was selected based on its balance between spatial proximity and grid connectivity.

Based on this scheme, the results of the global Moran’s I index reveal a significant positive spatial autocorrelation at the overall spatial scale (Moran’s I = 0.4053, p = 0.0010, z = 11.1120), indicating the presence of spatial dependence and clustering trends of sentiment scores within the study area.

**Fig 7:**
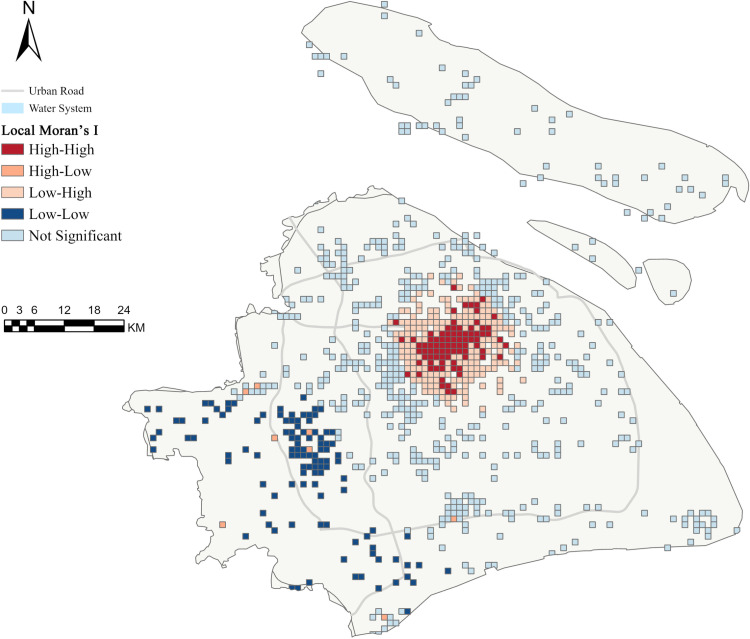
Results of Local Moran’s I analysis showing spatial clustering patterns of sentiment scores in Shanghai. Red areas indicate high-high clusters, blue areas represent low-low clusters, and light colors show spatially insignificant areas. Base map source: Natural Earth.

The local Moran’s I analysis further uncovers detailed geographic variations in sentiment experiences (Fig 7). Among the 880 analyzed units, 273 units (31.02%) exhibit statistically significant spatial autocorrelation (p < 0.05). High-high clustering areas are predominantly located at the intersections of Xuhui, Jing’an, Changning, and Huangpu districts, forming significant core clusters. In contrast, low-low clustering areas are mainly distributed in the southwestern part of the city, presenting a relatively dispersed spatial pattern.

To quantitatively characterize the spatial features of different clustering types, key metrics such as distance centrality and shape index were extracted (Table 5). The average distance centrality of high-sentiment clusters (1,263.54 meters) is significantly lower than that of low-sentiment clusters (1,847.92 meters), indicating that the former are more centrally located within the study area. The average shape indices of high- and low-sentiment clusters are 0.21 and 0.16, respectively, suggesting that high-sentiment areas exhibit more regular and compact spatial forms compared to low-sentiment areas.

**Table 5:** Spatial morphological characteristics of sentiment CLUSTERS.

Metric	High-Sentiment Clusters	Low-Sentiment Clusters
Average Distance Centrality	1,263.54 m	1,847.92 m
Average Shape Index	.21	.16

## Discussion

### The spatial organization mechanism of citywalk

This study, through quantitative analysis of multi-source data, reveals the spatial organization characteristics of Citywalk activities. The results indicate significant spatial clustering of functional diversity, transportation accessibility, and interaction intensity, which are closely associated with the distribution of emotional experiences.

From the perspective of the theory of social space production, Citywalk activities reflect the triadic dialectical relationship of “spatial practice, representations of space, and representational spaces” proposed by Lefebvre. This spatial clustering not only represents the material attributes of high-density POI areas but also highlights the deep coupling between diverse functions and social interactions. The findings show that areas with high sentiment scores (>300) often exhibit a higher degree of functional mix (e.g., Shannon entropy of 1.9–2.4) and stronger interaction intensity (e.g., index >100). This suggests that spatial production in the digital age transcends material dimensions, with social media playing a crucial role. These results align with prior research by Lindell, Jansson [42] on how geospatial media practices reproduce social positions through social media, while further extending the application of social space production theory in the context of virtual-physical interactions.

The theory of sense of place provides a complementary explanatory framework for the formation of emotional spaces. The significant correlation between sentiment scores and social media interaction intensity suggests that the formation of sense of place depends not only on the physical spatial environment but is also driven by virtual social interactions. This is consistent with findings from previous studies by Gatti and Procentese (15) regarding the multidimensional mechanisms underlying place attachment formation, further validating the role of social media in enhancing the sense of place. However, the spatial polarization of the interaction index (e.g., 75% of grid values below 1.043) indicates that this enhancement effect may be confined to core areas, with weaker interaction intensity in peripheral areas potentially leading to a diminished sense of place.

This study integrates dual perspectives of physical environment and digital social data. Through spatial analyses of functional diversity, transportation accessibility, and sentiment scores, it is observed that areas with high functional diversity are often associated with higher emotional experiences. This is consistent with conclusions from prior studies by Jin, Ge [43] on the contribution of functional diversity to spatial vitality. Moreover, this study reveals the critical role of social media interactions in the interplay between virtual and physical spaces, providing new empirical support for the theory of sense of place.

### Analysis of factors influencing citywalk sentiment experiences from a machine learning perspective

This study innovatively applies machine learning methods to analyze the sentiment experiences of Citywalk, an emerging urban leisure activity. By constructing an integrated analytical framework encompassing multi-source heterogeneous data (including high-resolution POI data, public transportation network data, and social media data), this work offers a novel perspective on the drivers of participation in Citywalk. Utilizing advanced algorithms such as SHAP feature importance analysis and segmented regression, the study systematically identifies key factors influencing participants’ emotional perceptions and their interaction patterns, thereby uncovering the complexity of individual-environment interactions.

The findings show that social media interaction intensity (interaction index) is the most significant factor explaining the variation in Citywalk sentiment scores (SHAP value: 4.9104). This quantitative result indicates that digital social behaviors may play a critical role in shaping new walking experiences. The high fit between the interaction index and sentiment scores (R² = 0.9422), with no clear threshold characteristics, suggests a sustained and gradual influence of online interactions on participants’ emotional feedback during Citywalk activities. This finding provides new empirical evidence for Lefebvre’s theory of the production of space [44], indicating that virtual social networks are becoming key components in shaping physical space experiences. Consistent with the insights of Fang, Yu [45], this study confirms that subjective perceptions of urban spaces in the digital age are increasingly influenced by online interpersonal networks. At the empirical level, the study highlights that evaluating Citywalk spatial quality cannot ignore the role of optimizing the digital social ecosystem. Urban planning should consider online interactions in design processes to optimize the interplay between virtual and physical environments, ultimately enhancing individual emotional experiences.

The number of Citywalk-related POIs emerges as the second most significant predictor (SHAP value: 2.4119), though its influence demonstrates a notable nonlinear pattern. Segmented regression results indicate a threshold effect: when POI density reaches 44.0553, its marginal utility in enhancing Citywalk sentiment experiences undergoes a sudden leap. This finding suggests that the relationship between POI density and emotional experiences may be nonlinear, diverging from the traditional notion that density straightforwardly enhances urban vitality. Instead, it indicates a more complex relationship between spatial elements and individual perceptions [46]. As Jacobs [47] noted, the vitality of streets depends on the intricate interplay of multiple elements. Using machine learning techniques, this study empirically examines this proposition from a nonlinear perspective using Citywalk sentiment as a case study. Identifying the POI density threshold not only deepens the understanding of Jacobs’ theory but also offers more flexible strategies for spatial optimization. Clarifying the threshold dynamics of key influencing factors can aid in devising phased environmental improvement plans, achieving simultaneous enhancements in emotional experiences and spatial quality within resource-constrained settings.

Compared to objective material space indicators, the explanatory power of social media interaction intensity for Citywalk sentiment scores is more prominent. This finding suggests that, relative to traditional urban morphological factors, social network interactions may serve as a critical entry point for understanding individual emotional experiences. This aligns with the recognized role of social networks in shaping urban spatial perceptions [48]. Furthermore, the study shows that social networks facilitate the flow of knowledge and information across physical boundaries, making urban spatial experiences more dynamic and diverse [49]. These results further indicate that virtual interpersonal networks are profoundly reshaping the perception of urban spaces, and traditional research focused solely on physical elements may need to expand its scope. By introducing detailed analyses of social media data, this study quantifies its association with sentiment scores, not only advancing the measurement of the relationship between online interactions and offline spatial experiences but also providing new perspectives on how virtual networks influence the mechanisms underlying urban spatial perceptions.

Grounded in the theory of social space production, this study integrates digital social behaviors with physical walking spaces, systematically analyzing the key factors influencing Citywalk sentiment experiences and their nonlinear effects using machine learning methods. Beyond traditional material spatial dimensions, this study incorporates virtual network interactions into the analytical framework, extending the application of social space production theory into the digital era. These findings provide not only new insights for optimizing urban spatial design and enhancing emotional experiences but also theoretical support for understanding how virtual networks reshape spatial perception mechanisms.

### Integrating emotional dimensions through the socio-spatial dialectic

The feature importance analysis of the machine learning model indicates that social media interaction intensity is the most significant factor explaining the emotional scores of Citywalk participants (SHAP value: 4.9104), surpassing objective indicators of the built environment such as POI density (SHAP value: 2.4119). This finding empirically validates the argument that social interactions play a crucial role in urban spatial experiences [50]. Furthermore, by employing nonlinear modeling approaches, the study quantitatively characterizes the complex relationship between interaction intensity and individual emotions. By introducing the interaction index, the research reveals that the generation of emotional spaces extends beyond the traditional “person-object” duality and evolves into a multidimensional construct shaped by “person-space-social media” interweavings [51].

This challenges the conventional paradigm of spatial quality assessment, where the focus on physical components alone often fails to comprehensively capture spatial performance, and neglecting human-place interactions mediated by digital platforms may result in evaluative biases. Participants’ preference for online interactions underscores that social networks have become critical mediators of perception and behavior, with virtual interactions influencing emotional experiences in physical spaces through virtual-to-real mapping [52]. Understanding virtual social trajectories is thus essential for gaining insights into individual spatial evaluations, indicating that human-centered space-making must be grounded in the dynamic coupling of online and offline interactions, incorporating digital social behaviors into planning considerations.

The nonlinear relationship between Citywalk-related POI density, functional diversity, and sentiment scores not only reveals the complexity of socio-spatial formation but also provides new empirical support for the theory of sense of place. The threshold characteristic of key factors (e.g., the inflection point of POI density at 44.0553) indicates that emotional attachment to places is not derived from a single factor but depends on a specific intensity and balance among environmental components [53, 54]. This finding aligns with the multidimensional mechanisms underlying the formation of sense of place, as prior studies have shown that place attachment results from the intertwined effects of physical environmental features, social interactions, and emotional and behavioral ties [55]. By leveraging big data analysis and machine learning methods, this study empirically elucidates the interplay and critical nodes of various influencing factors, thereby deepening the understanding of the mechanisms shaping the sense of place.

Although emotional attachment to places is rooted in subjective experiences, it is also embedded within socio-spatial dialectical processes [56]. Enhancing spatial quality requires moving beyond the determinism of physical elements, shifting toward place-making that centers on human perceptions. This entails focusing on the co-creation of experiences by users and physical settings to achieve breakthroughs at critical nodes.

The integration of geospatial big data opens new methodological pathways for studying sense of place. Combining such data with dynamic, fine-grained spatial behavior data addresses limitations of traditional spatial indicators in capturing complex emotional evaluations. The fusion of UGC data with conventional geographic information significantly enhances the ability to characterize subjective perceptions, as evidenced by the model’s R² of 0.8470. This highlights the substantial contribution of virtual interaction data to expanding frameworks for assessing sense of place. The utility of UGC lies not only in its direct documentation of emotional expressions but also in its ability to uncover the dynamic nature of human-place relationships.

In the digital age, virtual interactions and real-world experiences form mutually reinforcing feedback loops. Through activities such as social media comments, likes, and shares, users record and disseminate their experiences of places in virtual environments. These virtual interactions enhance the visibility and attractiveness of destinations, further optimizing real-world experiences and stimulating new virtual engagements [57]. The combination of sentiment analysis and machine learning enables the extraction of insights from large-scale heterogeneous data, paving the way for exploring individual-place emotional connections. Expanding the temporal and spatial dimensions of place assessment requires ongoing exploration of data integration, aiming to build comprehensive measurement systems encompassing multi-sensory experiences.

The spatial differentiation of sentiment scores revealed by local spatial autocorrelation analysis provides new empirical evidence for the geographic heterogeneity of sense of place [58]. The identification of hot and cold spots suggests that while individuals’ emotional experiences of places are rooted in subjective cognition, they also exhibit distinct spatial clustering patterns. The preference for central locations in high-sentiment areas (average distance: 1,263.54 meters) implies a potential association between centrality and stronger emotional attachment. This finding offers spatial layout references for human-centered place-making and informs strategies for optimizing activity nodes based on the spatial distribution of emotional scores.

### Limitations and future research directions

Despite its methodological and empirical value, this study has several key limitations. The first limitation concerns the issue of data representativeness. Although social media data provide rich emotional expression information, the user group of such platforms may exhibit biases in terms of age and socioeconomic status. Social media users are predominantly young individuals with higher digital literacy and social tendencies. This sampling bias may result in the findings not fully reflecting the emotional experience patterns of the broader urban population. Additionally, the spatial and temporal coverage of the data is limited. This study’s analysis is based on cross-sectional data from 880 grid units, lacking an in-depth examination of the temporal dimension, making it unable to effectively capture the seasonal variations and long-term evolutionary characteristics of emotional experiences.

At the methodological level, while the machine learning model demonstrates excellent predictive performance (R² = 0.8470), its “black box” nature still constrains the in-depth understanding of its predictive mechanisms. Although the SHAP method provides a certain level of interpretability, the decision-making processes of deep learning models remain difficult to fully elucidate [59]. The scale effect in spatial analysis also deserves attention. The spatial weight matrix, constructed based on a grid diagonal distance of 1,414.21 meters, may obscure spatial association patterns at finer scales, while potentially underestimating spatial spillover effects at larger scales.

The limitations of the theoretical framework are mainly reflected in the insufficient integration of sociocultural factors. Although the interaction index has been confirmed as the most important predictor (SHAP value: 4.9104), this indicator mainly reflects the quantitative characteristics of social behavior and is inadequate in capturing qualitative dimensions such as cultural identity and historical memory. Additionally, the study fails to adequately consider the impact of individual heterogeneity. Different social groups may have varied emotional responses to the same spatial environment, and this heterogeneity is not systematically examined within the current analytical framework.

There is also a clear deficiency in the identification of causal relationships. Although feature importance analysis and spatial statistical methods reveal the associative patterns between environmental factors and emotional experiences, whether these statistical relationships reflect true causal mechanisms still requires further validation.

Based on the aforementioned limitations, future research can be deepened in the following directions: First, in terms of data dimension expansion, diverse data sources such as mobile signaling data and field survey data can be integrated to enhance the representativeness and spatiotemporal coverage of samples. Additionally, incorporating individual characteristic data will help to understand emotional experience differences among different groups. Second, at the methodological level, more advanced interpretable artificial intelligence techniques can be explored to enhance model transparency; multi-scale spatial analysis methods can be developed to better capture association patterns across different spatial scales; causal inference methods, such as instrumental variables or quasi-experimental designs, can be applied to validate the causal validity of key findings. Furthermore, in terms of theoretical research, there is a need to strengthen the combination of qualitative and quantitative methods to deeply explore the mechanisms of sociocultural influences; conduct cross-city comparative studies to test the generalizability of conclusions; and build dynamic evaluation systems to track long-term evolutionary patterns.

## Conclusions

This study, using Shanghai’s Citywalk as a case study, innovatively incorporates social media data and machine learning methods into the spatial quality assessment of emerging leisure behaviors. By constructing a virtual-physical interaction evaluation framework, the study systematically explores the formation mechanism of urban walking emotional experiences. This analytical framework not only enriches the research perspective on the relationship between virtual behavior and physical space but also provides scientific evidence for optimizing walking environments.

The machine learning analysis of multi-source heterogeneous data reveals that social media interaction intensity is the dominant factor influencing Citywalk sentiment experiences, indicating that the formation of individual emotions is no longer confined to physical space but is also shaped by virtual social networks. This finding extends Lefebvre’s concept of “social space,” highlighting the value of digital behaviors in spatial studies. Moreover, the significant interaction effects between built environment factors and virtual social behaviors provide important insights for optimizing spatial design strategies.

Spatial statistical analysis further reveals that walking emotional experiences exhibit significant spatial clustering characteristics. This pattern of spatial differentiation reflects the profound impact of virtual-physical interaction on social space production. The centrality of location plays a critical role in shaping the emotional atmosphere of the city, indicating that human-centered space-making should be based on a networked layout of nodes and links, leveraging optimized resource allocation to amplify positive externalities.

In conclusion, this study demonstrates the expanded significance of the socio-spatial dialectic perspective in urban spatial quality assessment. The integration of digital social behaviors and machine learning methods broadens the application of sentiment analysis in the planning field, advancing the human-centered planning paradigm. It also builds a bridge for interdisciplinary dialogue between urban geography, spatial sociology, and emotional geography. Future research should focus on multi-sensory metrics, broaden spatiotemporal data dimensions, optimize artificial intelligence models, and promote interdisciplinary collaboration. By taking the dynamic nature of human-place relationships as a logical starting point and leveraging digital technologies to enable co-creation between users and environments, the ultimate goal is to achieve the creation of high-quality human-centric living environments.
